# Late Prevertebral and Spinal Abscess following Chemoradiation for Laryngeal Squamous Cell Carcinoma

**DOI:** 10.1155/2014/425724

**Published:** 2014-02-19

**Authors:** Jawad Hindy, Ilan Shelef, Yuval Slovik, Ben-Zion Joshua

**Affiliations:** ^1^Department of Otolaryngology & Head and Neck Surgery, Soroka University Medical Center, Ben Gurion University of the Negev, Rager Avenue, 84101 Beer-Sheva, Israel; ^2^Department of Radiology, Soroka University Medical Center, Ben Gurion University of the Negev, 84101 Beer-Sheva, Israel

## Abstract

*Objective*. Advanced primary supraglottic tumors (i.e., T3 or T4) have traditionally been treated surgically and postoperative radiotherapy. In the last 2 decades, some patients were treated with chemoradiation avoiding surgery. *Case Report*. We describe a 55-year old female who presented with respiratory distress and paraplegia seven years after treatment for a T3N0M0 supraglottic squamous cell carcinoma. CT scan showed prevertebral and intraspinal air descending from C4 to D3 vertebras. Epidural and prevertebral abscesses were confirmed by neck exploration. Necrosis was observed in the retropharyngeal, prevertebral, and vertebral tissues. *Conclusion*. Prevertebral and spinal abscess may result from chemotherapy and radiotherapy to the head and neck. Physicians caring for head and neck cancer patients treated with chemotherapy and radiation should be aware of this rare severe complication.

## 1. Introduction

Squamous cell carcinoma (SCC) is the most common malignant tumor of the larynx, responsible for between 85% and 95% of all laryngeal malignancies [1]. Common known risk factors for laryngeal cancer including smoking, alcohol, coffee consumption, and diesel exhaust fumes [2]. In Israel, Supraglottic SCC is less frequent than glottic SCC and account for 40% of laryngeal carcinomas. Majority of the lesions in supraglottic SCC are seen either on the epiglottis, false cords, or aryepiglottic folds. A number of therapeutic options are available for supraglottic SCC. Early-stage disease (stage I and II) is generally treated with single modality therapy, either surgery or radiotherapy (RT), whereas advanced disease (stage III and IV) is generally treated with combined modality therapy, either primary surgery followed by RT or chemoradiotherapy (CRT) [3], or primary CRT [4, 5]. Common complications of RT include dysphagia, aspiration, laryngeal edema, and chondronecrosis [5].

We present a case of an extremely rare complication of chemoradiation for supraglottic SCC.

## 2. Case Report

A 55-year old female was transferred to our hospital from another hospital suffering from respiratory distress and paraplegia.

Past medical history: 7 years prior to hospitalization she was treated for a supraglottic SCC (T3N1 M0) with CRT. The patient was treated to 72 Gy total using a 3-field arrangement with subsequent cone-down technique. Radiation was delivered via 6-MV photons generated by a Varian linear accelerator. The tumor was treated with 2 lateral fields limiting the cord to 46 Gy. The last 12 Gy were given with 9 MEV electrons. The lower neck was treated with an AP field to 50 Gy. The patient received 3 courses of concomitant Cisplatinum 100 mg/m^2^ and 5 FU 800/mg/m^2^ for 4 days. There were no serious side effects during her treatment. She had been followed elsewhere and was doing well without difficulty swallowing or breathing and there was no evidence of recurrent tumor.

Three days before arriving to our hospital she began complaining of neck and shoulder pain. Neurological examination showed also weakness in both of her legs; she did not get any specific treatment. A CT scan showed air bubbles at the prevertebral space at levels C7 to T1. The patient continued to deteriorate and later she lost the ability to move all four limbs and she began to complain of respiratory distress. At this stage she was transferred to our hospital. On the way in the ambulance she suffered from severe respiratory distress and low blood pressure 82/54 mmHg and was intubated. Upon arrival her vital signs were temperature 35°C, blood pressure 160/60 mmHg, pulse 70 beats/min, and saturation 98% with 50% oxygen supply. Her urinary output was 12 cc at the first 2 hours. She was sedated with Propofol and after receiving 1 push of 1000 cc normal saline, she was kept on a 100 cc/hour drip of normal saline plus a Noradrenalin drip at a rate of 5 *μ*g/minute for the rest of her hospitalization.

**Figure 1:**
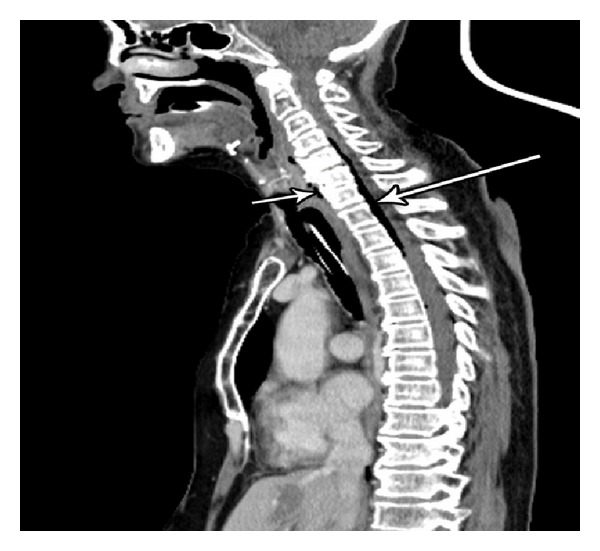
Postcontrast sagittal CT demonstrating air in the prevertebral space (short arrow) and spinal canal (long arrow).

**Figure 2:**
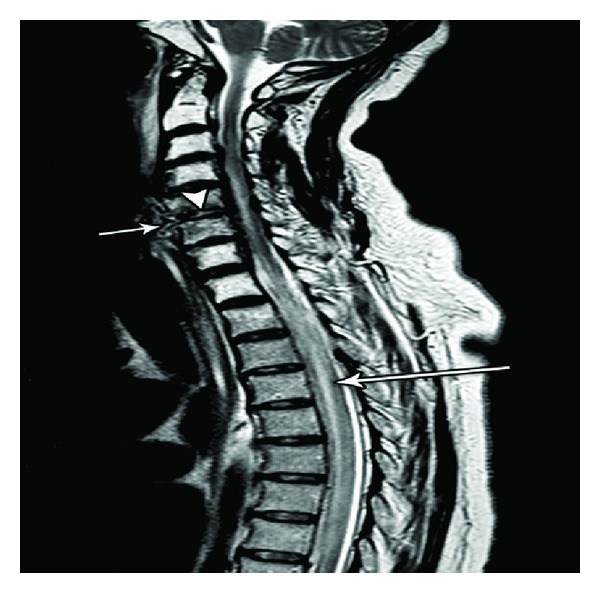
Sagittal T2 MRI demonstrating small collection in the prevertebral space (short arrow) with extension through C5-6 intervertebral disk space (arrow head) and edematous and abnormal high signal of the spinal cord (long arrow).

**Figure 3:**
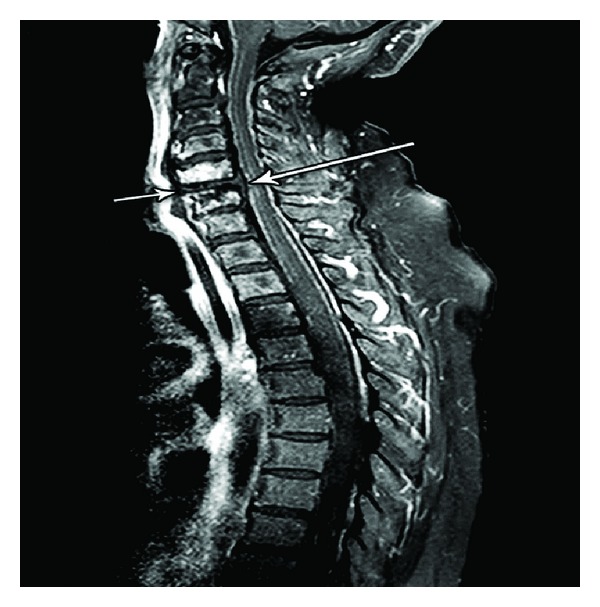
T1 MRI with fat suppression after IV Gadolinium in sagittal plane, demonstrating prevertebral collection (short arrow), with extension through the disk space into the epidural space (long arrow).

A repeat CT scan (Figure 1) and MRI (Figures 2 and 3) showed progression of the disease. An abscess with viscous perforation was suspected and exploration of the neck was performed. During exploration, we observed necrosis of the posterior pharyngeal fasciae and vertebrae which contained scant dishwash pus. The vertebrae which showed necrosis was debrided. Drains were left in place. A day later as she did not improve, neurosurgeons performed a re-exploration of the neck and drainage of the epidural abscess at levels C5-6, drains were left in place. Lumbar puncture did not reveal any bacterial growth or leukocytes in the CSF. Culture from the prevertebral pus was positive for Staph aureus. Antibiotics were started at the day of her admission, at first she was treated with IV Augmentin (1 gr × 3/day) along with IV Ceftazidime (2 gr × 3/day). After the prevertebral abscess bacterial grew Staph aureus, treatment was changed to IV Cloxacillin (3 gr × 4/day) plus IV Gentamicin (360 gr × 1/day). Biopsies which were taken from the necrotic tissues in the prevertebral fascia and the epidural abscess were free from malignant cells. Unfortunately, she continued to deteriorate and died 17 days after hospitalization at the ICU from sepsis.

## 3. Discussion

An epidural abscess of the spine is a rare disorder estimated to occur in 0.2–1.3 cases per 10,000 hospital admissions [6, 7]. Most of the patients with epidural spinal abscess have one or more associated clinical factors such as intravenous drug use, diabetes mellitus, spinal trauma, surgery, alcoholism, local or systemic source of infection, osteomyelitis, urinary tract infection, epidural analgesia, and nerve block [8–14]. Yet, no specific reports have mentioned laryngeal tumors as a predisposing factor causing epidural or spinal abscess. The most common bacterial infection is Staphylococcus aureus, comprising about two thirds of the cases [14–16]. Possible suggested mechanisms for the cervical epidural abscess in this case would be a direct extension through a fistula which was formed between the pharynx and the prevertebral fascia and was extending to the spine causing a chronic osteomyelitis; alternatively, it could be as a result of osteoradionecrosis of the vertebra causing chronic osteomyelitis. In both cases, it is difficult to state where the infection began since both the retropharyngeal wall and prevertebral and vertebral spaces were involved. Regardless, we believe that the most likely explanation for forming the abscess would be a sequela from the CRT treatment that our patient received. To the best of our knowledge, a prevertebral abscess many years after CRT for laryngeal SCC has not been reported yet.

The three most common symptoms of epidural spinal abscess are back pain, fever, and neurological deficit [14, 16, 17]. As in our patient who presented with respiratory distress and paraplegia, only a minority of the patients present with the whole classic clinical triad [10]. Aggressive treatment of the prevertebral abscess should be considered since such clinical picture is often fatal and the most sufficient management of it would be surgical drainage and debridement together with systemic antibiotics [6–9, 13, 16, 18].

Prognosis and final neurologic outcome are determined by the patient's neurologic status immediately before surgery [6, 7, 13, 18]. About 5% of the patients with spinal epidural abscess die, usually because of uncontrolled sepsis, evolution of meningitis, or other underlying illness [19]. In the case presented, chance of survival was very poor upon her arrival to our hospital since she presented with paraplegia. Moreover, the infection was widespread involving the posterior pharyngeal fasciae, the vertebrae, the epidural space, and the spine. Such a severe clinical picture decreased the chances of survival for our patient despite the aggressive treatment she received; eventually she died at the ICU from sepsis 17 days after hospitalization.

## 4. Conclusion

Prevertebral and spinal abscess may result from chemotherapy and radiotherapy to the head and neck. Physicians caring for head and neck cancer patients treated with chemotherapy and radiation should be aware of this rare severe complication.
